# miR-221-3p Drives the Shift of M2-Macrophages to a Pro-Inflammatory Function by Suppressing JAK3/STAT3 Activation

**DOI:** 10.3389/fimmu.2019.03087

**Published:** 2020-01-27

**Authors:** Lilian Quero, André N. Tiaden, Edveena Hanser, Julien Roux, Artur Laski, Jonathan Hall, Diego Kyburz

**Affiliations:** ^1^Experimental Rheumatology, University Hospital Basel, Basel, Switzerland; ^2^Department of Biomedicine, University of Basel, Basel, Switzerland; ^3^Bioinformatics Core Facility, Department of Biomedicine, University of Basel, Basel, Switzerland; ^4^Bioinformatic Core Facility, Swiss Institute of Bioinformatics, Lausanne, Switzerland; ^5^Department of Chemistry and Applied Biosciences, Institute of Pharmaceutical Sciences, ETH Zurich, Zurich, Switzerland

**Keywords:** innate immunity, macrophages (M1/M2), microRNAs, TLRs, RNA-seq, JAK3/STAT3, rheumatoid arthritis

## Abstract

**Objectives:** Macrophages are conventionally classified as pro-inflammatory (M1) and anti-inflammatory (M2) functional types. There is evidence for a predominance of macrophages with an inflammatory phenotype (M1) in the rheumatoid arthritis (RA) synovium. MicroRNAs (miRs) play a pivotal role in regulating the inflammatory response in innate immune cells and are found at dysregulated levels in RA patients. Here we explored miRs that tune the inflammatory function of M2-macrophages.

**Methods:** Expression profiles of miR-221-3p and miR-155-5p were analyzed in clinical samples from RA, other inflammatory arthritis (OIA), osteoarthritis (OA), and healthy donors (HD) by qPCR. *In vitro* generated macrophages were transfected with miR-mimics and inhibitors. Transcriptome profiling through RNA-sequencing was performed on M2-macrophages overexpressing miR-221-3p mimic with or without LPS treatment. Secretion of IL-6, IL-10, IL-12, IL-8, and CXCL13 was measured in M1- and M2-macrophages upon TLR2/TLR3/TLR4-stimulation using ELISA. Inflammatory pathways including NF-κB, IRF3, MAPKs, and JAK3/STAT3 were evaluated by immunoblotting. Direct target interaction of miR-221-3p and predicted target sites in 3'UTR of JAK3 were examined by luciferase reporter gene assay.

**Results:** miR-221-3p in synovial tissue and fluid was increased in RA vs. OA or OIA. Endogenous expression levels of miR-221-3p and miR-155-5p were higher in M1- than M2-macrophages derived from RA patients or HD. TLR4-stimulation of M1- and M2-macrophages resulted in downregulation of miR-221-3p, but upregulation of miR-155-5p. M2-macrophages transfected with miR-221-3p mimics secreted less IL-10 and CXCL13 but more IL-6 and IL-8, exhibited downregulation of JAK3 protein and decreased pSTAT3 activation. JAK3 was identified as new direct target of miR-221-3p in macrophages. Co-transfection of miR-221-3p/miR-155-5p mimics in M2-macrophages increased M1-specific IL-12 secretion.

**Conclusions:** miR-221-3p acts as a regulator of TLR4-induced inflammatory M2-macrophage function by directly targeting JAK3. Dysregulated miR-221-3p expression, as seen in synovium of RA patients, leads to a diminished anti-inflammatory response and drives M2-macrophages to exhibit a M1-cytokine profile.

## Introduction

Activated synovial macrophages are playing an essential role in generating the dysregulated conditions that promote chronic inflammation in rheumatoid arthritis (RA) (1–4). The interaction of activated macrophages and fibroblasts in the synovium is considered to drive the synovial hypertrophy eventually leading to the prominent clinical features of RA including the progressive destruction of articular cartilage and bone (5–8).

Macrophages are conventionally classified as M1 and M2 functional types. M1-macrophages display a pro-inflammatory/anti-pathogen function while M2-macrophages are segregated into several subtypes with either anti-inflammatory/resolving features or regenerative functions which support tissue homeostasis (9). Studies on macrophage function suggest that they display an exceptional plasticity, changing their function depending on the environmental conditions, their tissue-origin and cellular interactions (10, 11). Aberrant homeostatic conditions may therefore lead to a shift of the inflammatory cytokine profile in these cells (12, 13). Studies in RA patients and murine arthritis models reported an increased M1/M2-ratio in the synovium, which promoted osteoclastogenesis and skewed the adaptive immune response toward a Th1/Th17 function (2, 3, 14, 15). However, the mechanisms driving a dominant pro-inflammatory synovial macrophage population and their origin in RA are incompletely understood (16, 17). Regarding the development of an immunotherapeutical approach targeting synovial macrophages it is mandatory to address the regulatory mechanisms and underlying molecular pathways that cause the observed M1/M2-imbalance with increased production of pro-inflammatory cytokines by these cells.

Post-transcriptional regulation by microRNAs (miRs) represents an important mechanism controlling and modifying inflammatory responses (18, 19). Aberrant expression of miRs was reported to play a role in the development of many diseases such as cancer and autoimmune disorders (20, 21). Recently, miRs aroused increasing interest in the field of inflammatory arthropathy, especially for their use as molecular biomarkers (22) and as potential treatment targets (23, 24). Dysregulated miR expression in RA has been described (25, 26) and their relevance in RA was underscored for the example of miR-155 (27). The expression of miR-155-5p was found to be upregulated in different cellular compartments of patients with RA (28–30) and aberrant miR-155 expression was shown to be induced by Toll-like receptor (TLR) stimulation in macrophages (31) and to enhance inflammatory cytokine production (32). In addition, mice deficient for miR-155 did not develop collagen-induced arthritis, confirming the functional relevance of changes in expression of certain miRs in RA (33).

miR-221-3p has recently been reported in inflammatory conditions (34–37). To date, there are multiple descriptions of aberrant miR-221-3p expression predominantly in pathological conditions related to cancer (38–42) or obesity (43, 44). However, similar to miR-155-5p (31), miR-23a-3p and miR-27a-3p (45), miR-221-3p has also been shown to be regulated by TLRs (35).

TLRs are regulators of the inflammatory immune response (46) and TLR-signaling has been reported to be dysregulated in RA (47, 48). TLRs play an intrinsic role in the activation of innate immune cells in RA (49) and increased expression of TLR2/3/4 has been described in synovial tissue and cells (50–52) at early stages of the disease. CD14-positive cells from RA synovial fluid express more TLR2/4 and secrete increased levels of TNF-α and IL-8 compared to other inflammatory arthritis (OIA) (53). Recently, we and others demonstrated that TLR2-stimulation of M2-polarized macrophages resulted in a shift of the anti-inflammatory M2-profile to a pro-inflammatory M1-type (54, 55). The importance of TLR4-signaling in the propagation of joint inflammation and destruction in RA has been highlighted by *in vivo* studies demonstrating that TLR4-deficient mice or antibodies blocking TLR4-signaling exhibited less severe symptoms in collagen induced arthritis than control mice (56–58). Tissue damage and chronic infections can generate danger- and pathogen-associated molecular patterns (DAMPs and PAMPs) that are recognized by TLRs (59). Endogenous TLR-ligands such as HSP60, HMGB1, or DNA/RNA from necrotic cells have been described to be present in synovial fluid of RA patients (60, 61) and might therefore exuberantly activate synovial macrophages via TLRs.

Based on these findings, we conducted a study to investigate the role of miR-221-3p on the modulation of the inflammatory response in TLR-activated M1- and M2-macrophages.

## Materials and Methods

### Isolation, *in vitro* Differentiation and Stimulation of Monocyte-Derived Macrophages

Monocytes were isolated from peripheral blood of healthy donors (HD) (Blutspendezentrum, SRK beider Basel, Switzerland), patients with RA, other inflammatory arthritis (OIA: psoriatic- and spondyloarthritis) or osteoarthritis (OA) (Rheumatology Department, University Hospital Basel, Switzerland. RA as defined by the 2010 ACR/EULAR classification criteria). All blood donors gave informed consent. The study was approved by the Ethikkommission Nordwest- und Zentralschweiz (EKNZ), with the reference number EKNZ 2014-51. CD14^+^ monocyte isolation from peripheral blood mononuclear cells (PBMCs), differentiation into M1- or M2-macrophages using GM-CSF or M-CSF (Peprotech) and TLR-stimulation using 300 ng/ml Pam3CysSerLys4 (Pam3), 100 ng/ml ultrapure TLR4-specific LPS (LPS-EB) or 10 μg/ml polyinosinic:polycytidylic acid (PolyIC) (all InvivoGen, LabForce, Switzerland) was performed as previously described (55).

### miR Expression in Cultured Macrophages and Clinical Samples

Total RNA from cells (cultured macrophages, PBMCs, and CD14^+^ monocytes) or plasma, synovial fluid/tissue was isolated with miRNeasy Micro kit (Qiagen). 1 nM cel-miR-39 oligo (Qiagen/Exiqon) was added as spike-in control prior to RNA isolation from plasma and synovial fluid. Equal amounts of RNA were reverse transcribed with TaqMan miRNA Reverse Transcription/cDNA Synthesis Kit. qPCR was performed on a StepOnePlus using specific primers for mature miRs (all Applied Biosystems/ThermoFisher Scientific). Values of miR-221-3p, miR-27a-3p, and miR-155-5p were normalized to either RNU48, miR-103a-3p or miR-15b-3p (cultured macrophages), cel-miR-39 (plasma and synovial fluid), miR-16-5p, and miR-103a-3p (freshly isolated PBMCs/CD14^+^), miR-15b-3p (synovial tissue), and presented as 2^−ΔCT^ values by boxplot with min/max whiskers.

### miR Transfection Experiments

M1- and M2-macrophages were transfected with 10–25 nM of miRCURY LNA™ miR mimics of miR-221-3p, miR-27a-3p, or miR-155-5p or a respective control miR (Qiagen/Exiqon) using Lipofectamine2000 (ThermoFisher Scientific). In combined-transfection experiments, M2-macrophages were treated with miR inhibitors (Qiagen/Exiqon) or mimics for miR-221-3p and miR-155-5p at equal molarities. 24–48 h after transfection cells were stimulated with TLR ligands.

### RNA-Sequencing

M2-macrophages from 5 HD were cultured in 12 well plates with 540,000 cells/well and transfected with miR-221-3p mimic prior to stimulation with 100 ng/ml LPS for 8.5 h. Total RNA for the 20 resulting samples was isolated using miRNeasy Micro kit (Qiagen) and RNA quality was assessed with a Fragment Analyzer (Advanced Analytical).

RNA-seq library preparation (Illumina Truseq stranded kit) was performed at the Genomics Facility Basel of the ETH Zurich, where sequencing was performed on an Illumina NexSeq 500 machine. Single-end 81-mers reads were obtained and their quality was assessed with the FastQC tool (version 0.11.3).

Data were analyzed by the Bioinformatics Core Facility, Department of Biomedicine, University of Basel. Details on the analysis can be found in Supplementary File 1.

### ELISA

Following TLR-stimulation, supernatants were collected, and cytokine/chemokine release was measured by ELISA (IL-6, IL-8, IL-10, IL-12 ELISA kits from eBiosience/ Invitrogen/ThermoFisher Scientific; CXCL13 ELISA from R&D Systems). Values are presented either as concentration in pg/ml or as ratio.

### Immunoblotting

M2-macrophages were stimulated for 30 min to detect MAPK phosphorylation, for 1 h to assess nuclear shuttling of NF–κB and IRF3, or for 6/16 h for JAK3/STAT3 signaling. Whole cell or nuclear protein extracts were isolated as previously described (55). Equal amounts of protein were separated using SDS-PAGE gel and transferred onto PVDF membranes with a semi-dry blotting apparatus (TransBlotTurbo, BioRad). Proteins were stained as previously described (55) using the following primary antibodies (all 1:1,000; Cell Signaling Technology, BioConcept, Switzerland): rabbit anti-p38, rabbit anti-phospho-p38 (Thr180/Tyr182), mouse anti-ERK1/2, rabbit anti-phospho-ERK1/2 (Thr202/Tyr204), rabbit anti-SAPK/JNK, rabbit anti-phospho-SAPK/JNK (Thr183/Tyr185), mouse anti- NF–κB (p65), rabbit anti-IRF3, rabbit anti-JAK3, and rabbit anti-phospho-STAT3 (Tyr705), rabbit anti-β-Tubulin and rabbit anti-TBP. HRP-coupled goat anti-rabbit or goat-anti-mouse were used as secondary antibodies (1:2,000; Cell Signaling Technology, BioConcept, Switzerland), Chemiluminescence was detected using SuperSignal West Pico Plus (Pierce by ThermoFisher Scientific) and visualized by AGFA Curix 60 Developer system (Agfa, Belgium). Films were digitalized with an office scanner, images converted to 8-bit format and protein signal intensities were quantified using ImageJ software (version 2.0.0). Densitometric measurements of at least three different experiments were performed and represented as relative density ratio calculated from the protein of interest to its corresponding loading control.

### miR-221-3p Target Prediction in 3′UTR of JAK3

miR target prediction was performed with TargetScan 7.2 (www.targetscan.org) and miRanda/mirSVR (http://www.microrna.org/microrna/home.do) to identify potential binding sites of miR-221-3p in the 3'UTR of the human JAK3 mRNA.

### Luciferase Reporter Assay

JAK3 reporter plasmids were generated by cloning commercially synthesized target site inserts (Microsynth) into psiCHECK2 vector (no. C8021, Promega). Sequences of inserted target sites were as follows:

JAK3 Site-A 5′-3′:

CCTCCACTTCAGCCAGGACTCGAGGAGTTTCCTGTCTTATTTCCAATGGGGACATTCATGTAGCTTTTTTTTTTGCGGCCGCTGAGTCTTCGGACCTCGC

JAK3 Site-B 5′-3′:

CCTCCACTTCAGCCAGGACTCGAGGGCATGAGCCACTGCGCCCGGCCCTCATGTAGCTTTAAATGTATGATCTGGCGGCCGCTGAGTCTTCGGACCTCGC

Luciferase reporter assays were performed with 1.5 × 10^4^ HEK293T cells (ATCC® CRL-3216™) seeded into 96-well plates in DMEM (Gibco, Invitrogen) supplemented with 10% FBS (Gibco, Invitrogen). After 8 h, cells were transfected with RNA in technical triplicates, using Lipofectamine 2000 (ThermoFisher Scientific) according to the manufacturer's protocol. RNA transfections have been done with increasing concentrations (0, 2.5, 10, 40 nM) of miR-221-3p mimic (Qiagen/Exiqon), positive control RNA (siRenilla acting against Renilla luciferase) and negative control RNA (double-stranded RNAs carrying randomized base pairs) (62). After 24 h following RNA transfection, cells were transfected with 20 ng/well of indicated reporter plasmid, using JetPEI (10110N, Polyplus Transfection] according to the manufacturer's protocol. After 48 h following the plasmid transfection, luciferase readout was conducted using the Dual-Glo Luciferase Assay System (Promega) according to the manufacturer's protocol. Luminescence readout was performed on a microtiter plate reader (Mithras LB940, Berthold Technologies). Readout values were normalized to the firefly luciferase counts and additionally to the corresponding values obtained from 0 nM treatment.

### JAK Inhibition Experiments

M1- and M2-macrophages were treated with 5, 1, 0.1, and 0.01 μM selective JAK inhibitors FM-381 or Tofacitinib in DMSO (Sigma Aldrich/Merck) 1 h prior to TLR4-stimulation. DMSO-only at adjusted concentrations was used as negative control.

### Statistical Analysis

Statistical analyses were carried out using GraphPad Prism 7. Parametric analysis of normally distributed data was either performed by paired *t*-test or using the Friedman-test with Dunn's multiple comparisons test. Non-parametric data were analyzed by Wilcoxon matched-pairs signed ranked test or carried out by ordinary one-way ANOVA with Greenhouse-Geisser correction for multiple group analysis. We considered *p* < 0.05 significant. Data are presented as either boxplot with min/max whiskers (qRT-PCR) or by the mean ± standard deviation (SD) (ELISAs, Western Blot quantification).

## Results

### Expression of miR-221-3p and miR-155-5p Is Increased in Clinical Samples of RA Patients

**Figure 1:**
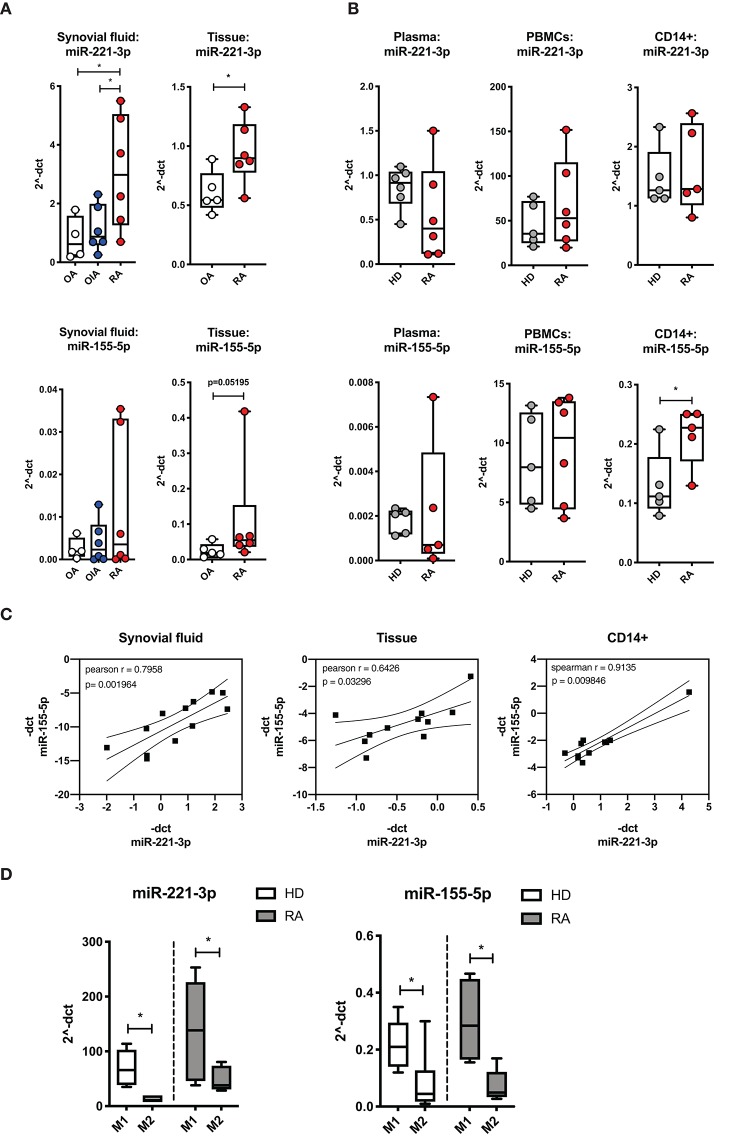
Comparison of miR-221-3*p* and miR-155-5*p* expression levels in clinical samples from healthy donors (HD), patients with rheumatoid arthritis (RA), other inflammatory arthritis (OIA) and osteoarthritis (OA). **(A)** miR expression levels in the synovial fluid of RA patients were compared to patients with OIA (psoriatic- and spondyloarthritis) and OA, and miR expression in synovial tissue was compared between biopsy samples from RA and OA patients. **(B)** miR expression levels were measured in plasma, PBMCs and isolated CD14^+^ cells from the peripheral blood of RA patients and HD. miR expression was measured by qRT-PCR and normalized to control miRs (miR-16-5*p*, miR-103-5*p*, miR-15b-5*p*, or spike-in cel-miR-39) and expressed as 2^−ΔCT^ by boxplot with min/max whiskers. *N* = 4–6, *p < 0.05. **(C)** −ΔCT level of miR-221-3*p* was plotted against miR-155-5*p* level from synovial fluid, tissue and CD14^+^ cells from blood and the Pearson or Spearman correlation and *p*-values were computed. Data are plotted with regression coefficients (solid line) and 95% confidence intervals (broken lines). **(D)** Expression levels of tested miRs were compared in M1- and M2-macrophages generated from CD14^+^ cells of PBMCs from HD or RA patients and presented as 2^−ΔCT^ by boxplot with min/max whiskers. *N* = 3–6, *p < 0.05.

We first examined the unstimulated expression levels of miR-221-3p and miR-155-5p in clinical samples of RA patients and compared them to OIA, OA or HD (Figure 1). We measured elevated levels of miR-221-3p in synovial fluid and synovial tissue from RA compared to OIA and OA (Figure 1A, upper panel), while there were no significant differences in plasma, PBMCs or CD14^+^ monocytes compared to HD (Figure 1B, upper panel). In line with earlier reports, miR-155-5p expression was increased in synovial fluid and tissue of RA patients (Figure 1A, lower panel), as well as in CD14^+^ monocytes but no difference was detected in plasma and in PBMCs from RA compared to HD (Figure 1B, lower panel). Furthermore, across all patients, miR-221-3p expression levels positively correlated with miR-155-5p measured in synovial fluid, tissue as well as CD14^+^ cells from blood (Figure 1C). To verify that the increased levels of miR-221-3p and miR-155-5p in RA synovium are not due to generally increased miR-levels in inflammatory conditions, we investigated miR-27a-3p as control. miR-27a-3p is a member of the miR-23a-miR-27a-miR24a cluster and was reported to be involved in the inflammatory response of macrophages (63) and inflammatory diseases such as PsA (45). In contrast to the miR-221-3p and miR-155-5p expression profiles, we measured no changes of miR-27a-3p in RA synovial fluid and tissue compared to OA (Figure S2A) but significantly lower levels in RA plasma and CD14^+^ cells (Figure S2B). Furthermore, there was no correlation detectable of the expression level of miR-27a-3p to the other investigated miRs in synovial tissue (Figure S2C). In summary, the expression profile of miR-221-3p displayed an overall similar pattern to miR-155-5p, with elevated levels in synovial fluid and tissues of RA patients, the primary site of inflammation in RA.

As a dysregulated balance of M1/M2-macrophage populations has been proposed to contribute to the chronic inflammation in the RA synovium (2, 14), we compared the endogenous expression level of miR-221-3p and miR-155-5p in differentiated M1- to M2-macrophages (Figure 1D). Interestingly, we detected a higher expression level of both miRs in M1- compared to M2-macrophages, pointing toward a pro-inflammatory feature of these two miRs. This M1-specific upregulation of miR-221-3p and miR-155-5p was measured in cells from HD as well as RA. By contrast, no significant difference was detected in miR-27a-3p expression level across both macrophage types (Figure S2D).

### TLR4-Stimulation Regulates Expression of miR-221-3p, miR-155-5p, and miR-27a-3p in Macrophages

**Figure 2:**
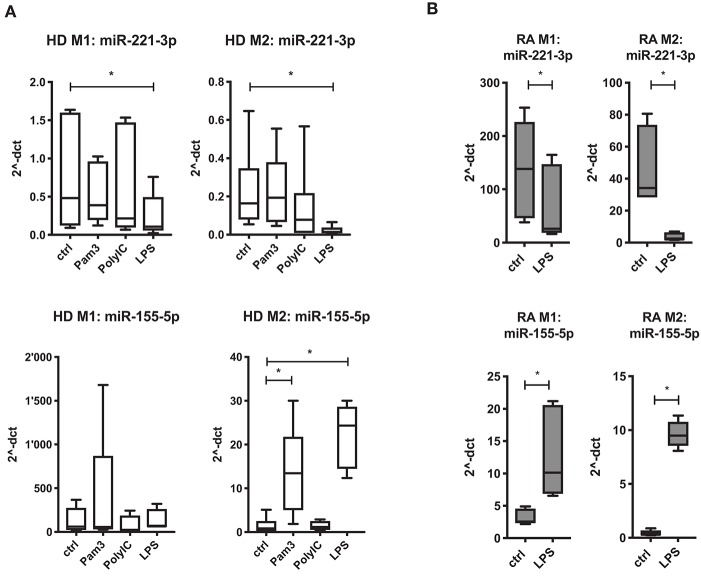
Regulation of miR 221-3*p* and miR-155-5*p* in M1- and M2-macrophages by TLR ligands. Macrophages differentiated from CD14^+^ cells from **(A)** HD or **(B)** RA blood were stimulated with either 300 ng/ml Pam3 (TLR2), 10 μg/ml PolyIC (TLR3) or 100 ng/ml LPS (TLR4) for 24 h. Changes in miR expression levels were measured by qRT-PCR. Values were normalized to RNU48, miR-103a-3*p*, or miR-15b-5*p* and presented as 2^−ΔCT^ by boxplot with min/max whiskers. *N* = 3–6, *p < 0.05 compared to untreated cells (ctrl).

miR-155-5p and miR-27a-3p have been reported to be regulated by TLRs in murine and human macrophages (31, 63). Here we investigated the regulatory effect of TLR signaling on the three selected miRs in human M1- and M2-macrophages. Interestingly, TLR4-stimulation by LPS caused a significant downregulation of miR-221-3p and miR-27a-3p, more prominently in M2- than in M1-macrophages (Figure 2A; Figure S2E). In contrast, TLR4 activation led to a prominent upregulation of miR-155-5p, but only in M2-macrophages. TLR2 ligand Pam3 did not significantly change the expression profile of miR-221-3p and miR-27a-3p but upregulated miR-155-5p, again only in M2-macrophages. TLR3 activation via PolyIC treatment caused a slight trend to downregulate miR-221-3p and miR-27a-3p in M2-macrophages, but did not affect miR-155-5p.

Since TLR4-stimulation displayed the most prominent effects on all three investigated miRs, we calculated the relative change of miR expression upon LPS-stimulation in macrophages generated from RA patients (Figure 2B; Figure S2F). We measured a similar regulatory effect by TLR4 activation on the respective miRs in M2-macrophages with the exception that miR-155-5p was also significantly increased in M1-macrophages of RA patients.

### Increased miR-221-3p Expression Alters the Cytokine and Chemokine Secretion Profile of TLR4-Stimulated M2-Macrophages

**Figure 3:**
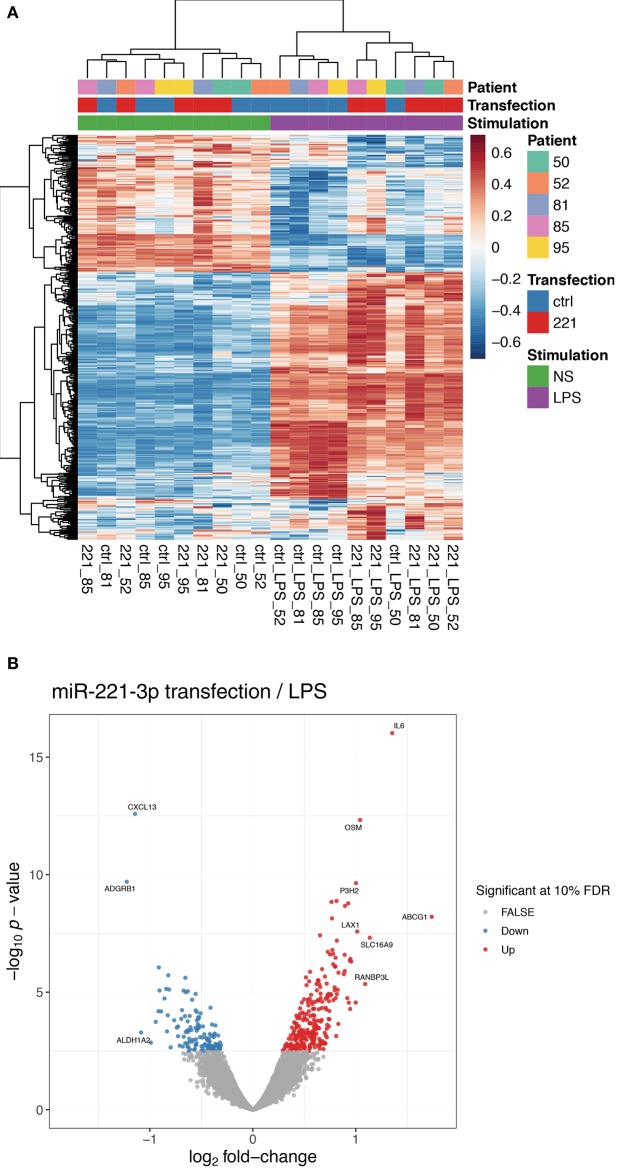
Gene expression profile of M2-macrophages transfected with miR-221-3*p* mimic plus/minus LPS stimulation. **(A)** RNA-seq was performed of *in vitro* generated M2-macrophages transfected with control miR (ctrl) or miR-221-3*p* mimic (221) and stimulated with LPS for 8.5 h or left untreated (NS). The heatmap displays the centered and scaled gene expression levels of genes differentially expressed between miR-221-3*p* transfected and control cells in LPS-stimulated conditions. To better visualize the differences across transfected and control cells, the expression levels were corrected for donor-specific effects (see RNA-seq methods in the Supplementary File 1). **(B)** Volcano plot showing the differentially expressed genes between cells transfected with miR-221-3*p* mimic and control cells in LPS-stimulated cells. Significant genes are colored in red (up-regulated) and blue (down-regulated). For clarity, only genes with an absolute log2-fold change larger than 1 are annotated.

As we detected an increased level of miR-221-3p in clinical samples of RA patients and as we measured a lower endogenous expression level of miR-221-3p in anti-inflammatory M2-macrophages compared to pro-inflammatory M1-macrophages of HD as well as RA patients, we were interested in the effect of dysregulated increased miR-221-3p levels in M2-macrophages. We therefore performed RNA-sequencing on M2-macrophages transfected with miR-221-3p mimics or a control mimic. Furthermore, as miR-221-3p was downregulated by TLR4-activation under physiological conditions, we also challenged M2-macrophages transfected with miR-221-3p mimics or a control mimic upon LPS treatment, thereby counteracting the TLR4-dependent repression on miR-221-3p. As expected, macrophage activation by LPS-stimulation had an overall prominent impact on the transcriptome of M2-macrophages as most of the genes were differentially regulated and only a few genes were not affected (Figure 3A, see also Figure S1; Table S1). Transfection of miR-221-3p mimic in unstimulated cells showed only mild effects (Table S2). Instead, M2-macrophages transfected with miR-221-3p mimics followed by LPS-stimulation showed significantly higher expression of 255 genes, while 132 genes were downregulated (Table S3). The list of candidate genes regulated by miR-221-3p under LPS condition included several inflammatory cytokines, chemokines, and their receptors. Thus, the levels of pro-inflammatory IL-6 or oncostatin M (OSM) were both upregulated upon miR-221-3p mimic transfection while LPS-induced expression of CXCL13 was downregulated (Figure 3B).

**Figure 4:**
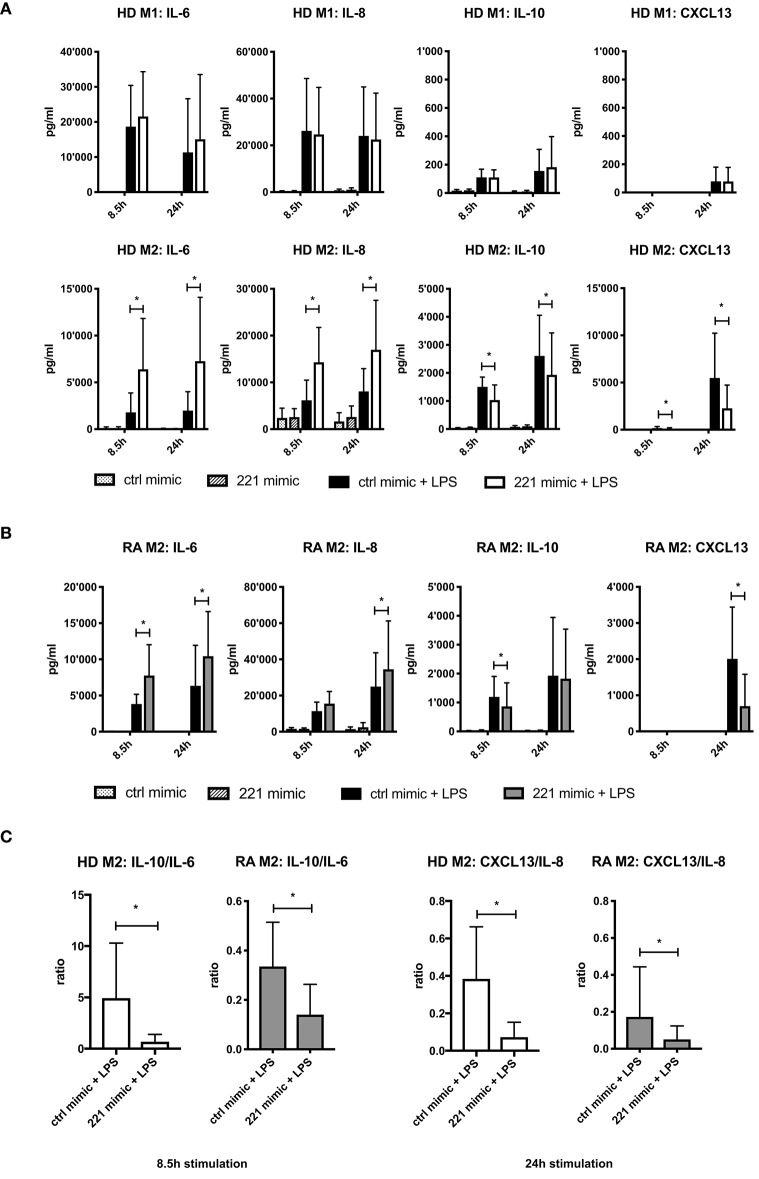
Effects of miR-221-3*p* mimics on cytokine and chemokine release in M1- and M2-macrophages. **(A)** M1- and M2-macrophages differentiated from CD14^+^ cells from HD blood were transfected with mimic of miR-221-3*p* (221 mimic) or a respective control miR (ctrl mimic) and stimulated with 100 ng/ml LPS or left untreated. Secreted cytokines and chemokines were measured after 8.5 and 24 h by ELISA and values are expressed as mean ± S.D. *N* = 6–10, *p < 0.05. **(B)** M2-macrophages differentiated from CD14^+^ cells from blood of RA patients were transfected with mimic of miR-221-3*p* (221 mimic) or a respective control miR (ctrl mimic) and stimulated with 100 ng/ml LPS or left untreated. Secreted cytokines and chemokines were measured after 8.5 and 24 h by ELISA and values are expressed as mean ± S.D. *N* = 6–11, *p < 0.05. **(C)** Anti-inflammatory activity of generated M2-macrophages from HD or RA patients was calculated after 8.5 and 24 h LPS-stimulation by the ratio of secreted cytokines IL-10 to IL-6 or chemokines CXCL13 to IL-8. Values are expressed as mean ± S.D. *N* = 6–11, *p < 0.05.

We next validated the differential expression patterns of IL-6, CXCL13 (among most affected by miR-221-3p in RNA-seq results), IL-8, and IL-10 (specific for either M1 or M2, respectively) at the protein level in M2-macrophages derived from HD (Figure 4). Additionally, we also assayed M1-macrophages derived from the same donors. We verified the stability of the cytokine/chemokine profile over time by measuring secreted protein levels after 8.5 and 24 h following LPS-stimulation. In cells transfected with mimic control, LPS-stimulation promoted pro-inflammatory IL-6 and IL-8 secretion mainly by M1-macrophages whereas anti-inflammatory IL-10 and CXCL13 were predominantly secreted by M2-macrophages. In LPS-stimulated M1-macrophages there was no significant difference in cytokine/chemokine levels between miR-221-3p mimic and control cells (Figure 4A, upper panel), whereas M2-macrophages transfected with miR-221-3p mimic displayed a significantly higher secretion of pro-inflammatory IL-6 and IL-8 and a significantly lower secretion of anti-inflammatory IL-10 and CXCL13 than control cells (Figure 4A, lower panel). Such differences in IL-6, IL-8 and IL-10 were not observed in M2-macrophages stimulated with TLR2- or TLR3-ligands (Figure S3). Interestingly, miR-221-3p mimic transfection in unstimulated conditions did not affect the levels of these proteins, suggesting a molecular coupling of miR-221-3p and TLR4-signaling. In contrast, transfection of M1- and M2-macrophages with miR-27a-3p mimics caused no significant changes of the selected cytokines and chemokines upon LPS treatment (Figures S2G/H).

TLR4-stimulated M2-macrophages generated from RA blood displayed similar differences on cytokine/chemokine secretion upon miR-221-3p mimic transfection compared to control cells (Figure 4B). Taken together, elevated levels of miR-221-3p changed the cytokine/chemokine secretion profile of TLR4-stimulated M2-macrophages toward a more pro-inflammatory phenotype with a decreased cytokine-ratio of IL-10/IL-6 and decreased chemokine-ratio of CXCL13/IL-8 (Figure 4C).

### miR-221-3p Affects JAK/STAT Signaling in M2-Macrophages by Regulating JAK3 Protein Expression

**Figure 5:**
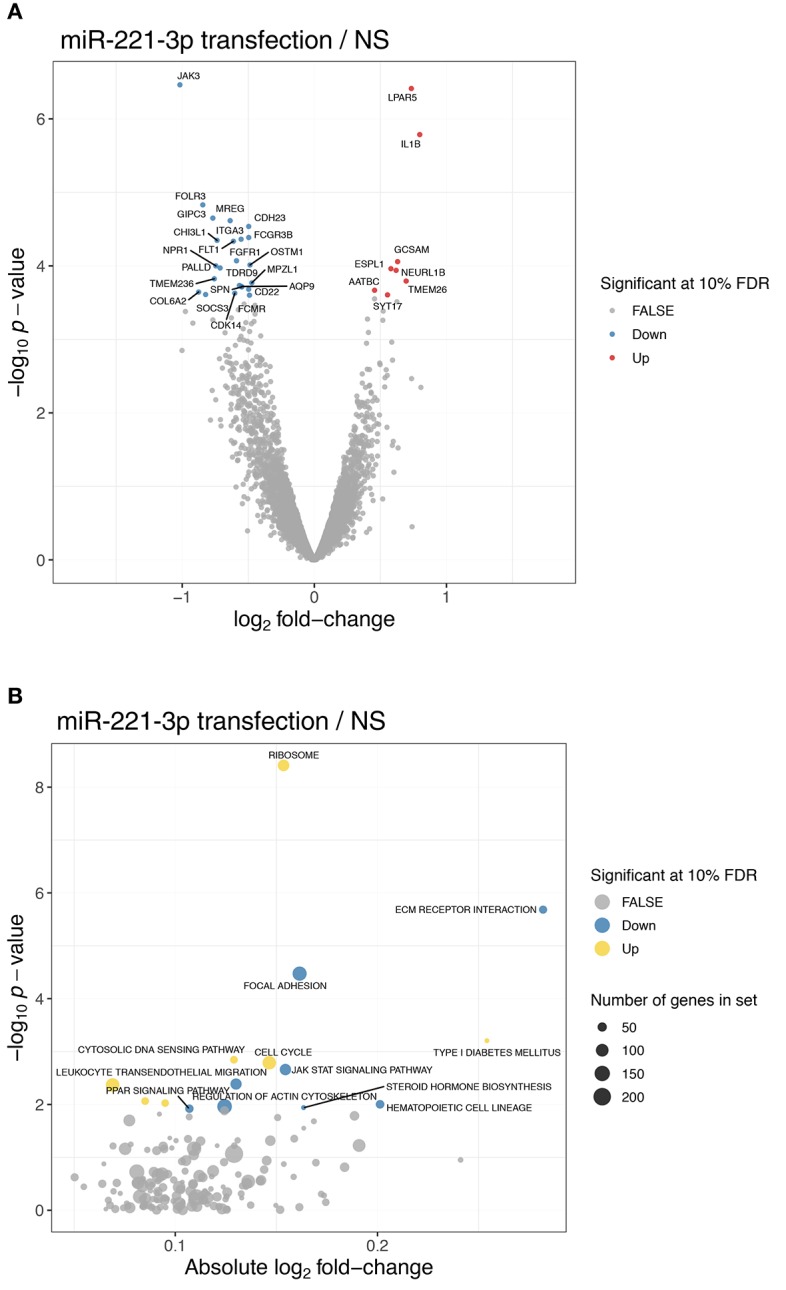
JAK3 as top downregulated gene of unstimulated M2-macrophages transfected with miR-221-3*p*. **(A)** Volcano plot showing the differentially expressed genes between cells transfected with miR-221-3*p* mimic and control cells in untreated conditions. Significant genes are colored in red (up-regulated) and blue (down-regulated). **(B)** Significantly enriched KEGG pathways among differentially expressed genes upon transfection in untreated conditions after gene set enrichment analysis. The absolute log2 fold-change (x-axis) is shown relative to the significance (y-axis). Significant pathways are colored in yellow (up-regulated pathway) and blue (down-regulated pathway). The dot size indicates the number of genes annotated in each pathway.

To reveal the pathways playing a role in the effects seen by miR-221-3p mimics, we screened the RNA-seq data for the strongest downregulated genes in unstimulated cells. JAK3 was the most significantly reduced gene by miR-221-3p mimic (Figure 5A). Related to this pattern, gene set enrichment analysis (GSEA) confirmed that the KEGG pathway “JAK-STAT signaling” was also significantly downregulated (Figure 5B). The JAK-STAT pathway represents an essential component of cytokine signaling in innate immune cells and has been shown to be targeted by TLR4 (64). Furthermore, JAK3 was reported to regulate IL-10 release (65) and STAT3 is supporting M2-specific macrophage polarization (12, 66).

**Figure 6:**
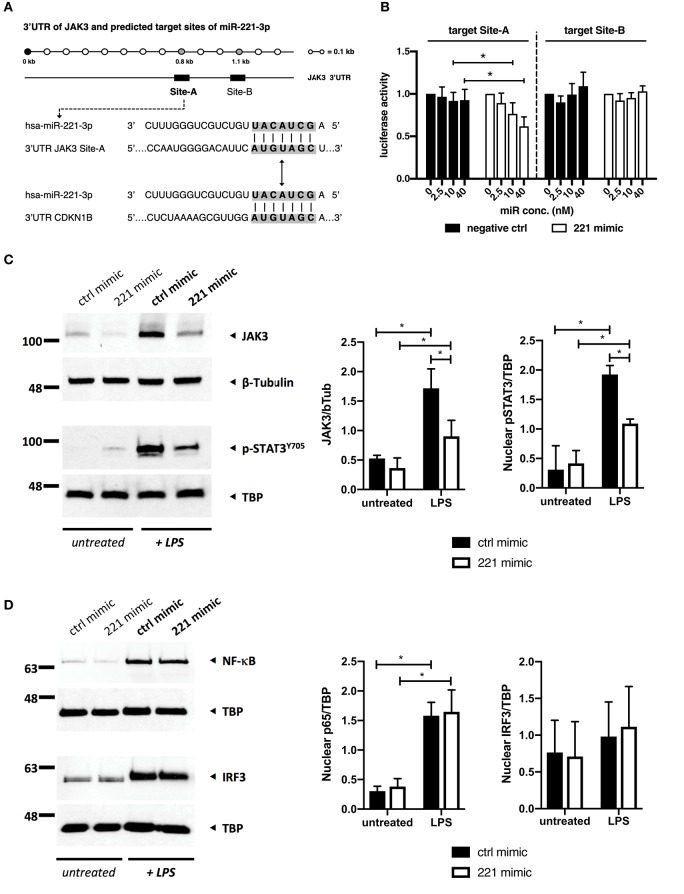
miR-221-3*p* is directly interfering with JAK3/STAT3 signaling in TLR4-stimulated M2-macrophages. **(A)** Location of two predicted miR-221-3*p* targets, Site-A and Site-B, within the 3'UTR region of the human JAK3 mRNA. Sequence of Site-A contains seed match region identical to already established miR-221-3*p* target on CDKN1B gene (gray shading). **(B)** Luciferase reporter assay of constructs carrying JAK3 Site-A and JAK3 Site-B target sites, co-transfected with increasing concentration of miR-221-3*p* (221 mimic) or RNA duplex carrying randomized base pairs (negative ctrl). Luciferase activity represents Renilla/firefly values normalized 0 nM and is expressed as mean ± S.D. *N* = 3–8, *p < 0.05. Differentiated M2-macrophages derived from CD14^+^ cells of HD blood were transfected with miR-221-3*p* mimic (221 mimic) or a respective control miR (ctrl mimic) and stimulated with 100 ng/ml LPS for **(C)** 16 h and **(D)** 1 h or left untreated. Protein levels of **(C)** JAK3 and phosphorylated STAT3 and nuclear shuttling of **(D)** *p*65 (NF-κB) and IRF3 were analyzed by SDS-PAGE and visualized by immunoblotting. TBP and β-Tubulin were used as loading controls for nuclear protein extract or whole cell lysates, respectively. Shown are representative images and quantification of *N* = 3–5. Quantification of protein expression was measured using ImageJ software and represented as a relative density ratio (protein of interest to loading control). Values are expressed as mean ± S.D. *N* = 3–5, *p < 0.05.

To examine whether JAK3 serves as a direct target of miR-221-3p in macrophages, we performed a miR target site prediction analysis using two distinct algorithms, TargetScan and miRanda/miRSVR. Both prediction tools independently identified two potential miR-221-3p binding targets (Site-A and Site-B) within the 3'UTR of JAK3 gene (Figure 6A). Interestingly, these sites were conserved only among hominids and not present in rodents (mice or rats). In addition, the same binding site sequence is present in CDKN1B (p27), a confirmed direct target of miR-221-3p (67). To investigate whether predicted target sites are involved in regulation of JAK3 gene, we constructed two luciferase vectors which got tested in luciferase reporter assay for inhibition by miR-221-3p (Figure 6B). While Site-B did not seem to be significantly regulated by miR-221-3p, Site-A displayed significant and dose-dependent downregulation while being subjected to miR-221-3p activity.

Given the results from the luciferase reporter assay supporting a direct interaction of miR-221-3p with JAK3 we examined the effect of miR-221-3p mimics on the JAK/STAT signaling in TLR4-activated M2-macrophages. We first found that TLR4-activation by LPS induced JAK3/STAT3 signaling in M2-macrophages as we detected significantly increased JAK3 protein as well as phosphorylated Y705-STAT3 (pSTAT3) in the nucleus (Figure 6C). In contrast, administration of miR-221-3p mimics significantly reduced JAK3 and pSTAT3 protein levels, confirming the interference of miR-221-3p with the JAK3/STAT3 pathway.

To check the possibility that TLR4 signaling *per se* was affected by miR-221-3p mimics we investigated distinct pathways downstream of TLR4. The MyD88-dependent pathway leads to an activation of NF-κB (p65) and MAPKs, whereas the TRIF-dependent pathway involves activation of IRF3. Transfection of M2-macrophages with miR-221-3p mimic did not affect these pathways, as we did not see any difference in nuclear shuttling of p65 and IRF3 (Figure 6D), and no change in the phosphorylation status of the MAPKs ERK1/2, JNK, or p38 (Figure S4) following the activation with LPS.

In summary, we identified JAK3 as new direct target of miR-221-3p and confirmed that forced high levels of miR-221-3p following TLR4-activation were suppressing JAK3/STAT3 signaling in M2-macrophages.

### Pharmacological Inhibition of JAK3 Alters the Cytokine Profile of TLR4-Stimulated M2-Macrophages

**Figure 7:**
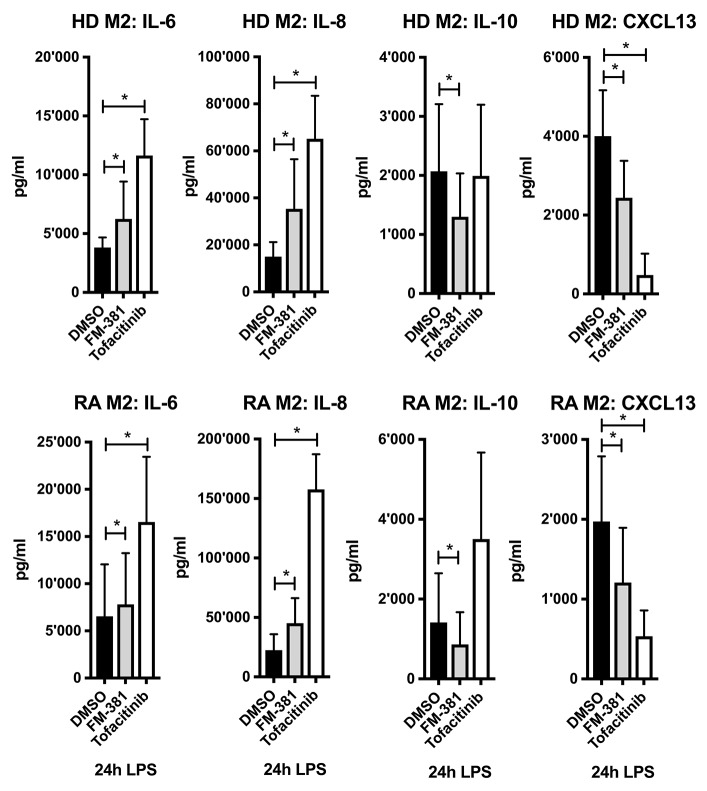
JAK3 activity was inhibited in generated M2-macrophages from blood of HD or RA patients using two selective JAK inhibitors FM-381 and Tofacitinib. Cells were treated 1 h before LPS stimulation with 1 μM JAK inhibitor or DMSO control. Cytokine and chemokine secretion were measured by ELISA after stimulation with LPS for 24 h. Values are expressed as mean ± S.D. *N* = 3–8, *p < 0.05.

To investigate whether the altered cytokine/chemokine profiles in M2-macrophages could be the result of reduced JAK3/STAT3 signaling, we inhibited JAK3 function using the selective inhibitors FM-381 and Tofacitinib. We measured the impact of JAK3 inhibition on the cytokine/chemokine profile of LPS-stimulated M1- and M2-macrophages derived from HD. We found that the JAK3-selective inhibitor FM-381 exerted similar effects on IL-6, IL-8, IL-10, and CXCL13 secretion by M2-macrophages (Figure 7, upper panel) as transfection with miR-221-3p mimics (Figure 4A, lower panel). Notably, Tofacitinib as a less selective pan-JAK-inhibitor did not alter IL-10 secretion, but exhibited more pronounced effects on IL-6, IL-8, and CXCL13 compared to FM-381, possibly due to an involvement of other JAK family members in the regulation of these cytokines/chemokines. Similar results were obtained by using M2-macrophages generated from RA blood (Figure 7, lower panel). Both inhibitors exhibited a dose-dependent effect on the measured cytokines/chemokines (Figure S5, right panel). In contrast to M2-macrophages, we detected the opposite effect of JAK3 inhibition in M1-macrophages, as both inhibitors suppressed pro-inflammatory IL-6 secretion in a dose-dependent manner (Figure S5, left panel). In summary, selective JAK3 inhibition by FM-381 in TLR4-activated M2-macrophages led to similar changes as transfection of miR-221-3p mimics.

### Dominant Impact of miR-221-3p in TLR4-Activated M2-Macrophages Co-transfected With miR-221-3p/ miR-155-5p Mimics

**Figure 8:**
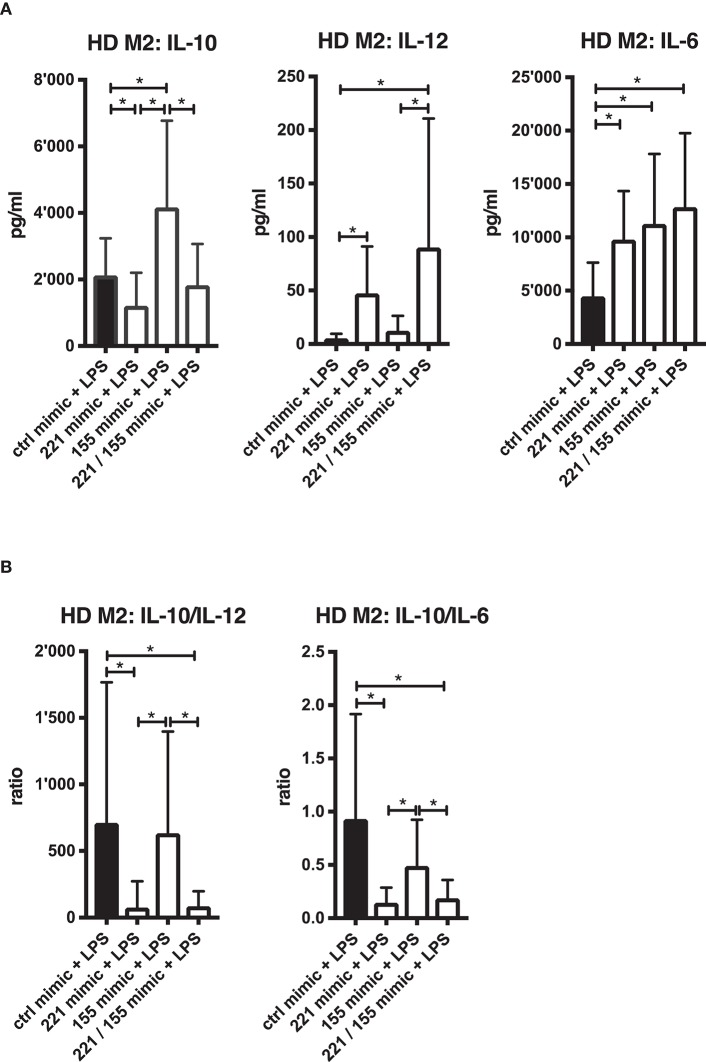
Effects of combined miR-155-5*p*/miR-221-3*p* mimics on pro- and anti-inflammatory cytokine secretion in TLR4-stimulated M2-macrophages. Cells were differentiated from CD14^+^ cells derived from HD blood. **(A)** M2-macrophages were transfected with mimics of miR-155-5*p* and miR-221-3*p*, either alone (155 or 221 mimic) or combined (155/221 mimic) and a control miR (ctrl mimic). M2-macrophages were then stimulated with 100 ng/ml LPS or left untreated. Secreted cytokines were measured after 24 h by ELISA and values are expressed as mean ± S.D. *N* = 15–18, *p < 0.05. **(B)** Anti-inflammatory activity of generated M2-macrophages transfected with the mentioned mimic combination setup was calculated after 24 h LPS-stimulation by the ratio of secreted cytokines IL-10 to IL-12 or IL-10 to IL-6. Values are expressed as mean ± S.D. *N* = 15–18, *p < 0.05.

miR-221-3p and miR-155-5p are both present at elevated levels in synovial compartments of RA patients (Figure 1) and miR-155-5p has been shown to play a prominent role in the development of arthritis (27). We therefore investigated the effect of combined miR-221-3p/miR-155-5p mimic transfection on the inflammatory response of M2-macrophages upon TLR4-stimulation (Figure 8). To evaluate a shift in macrophage-type function we measured IL-10 as a marker for anti-inflammatory M2 function, IL-12 and IL-6 for pro-inflammatory M1 function. Transfection of M2-macrophages with miR-155-5p mimics alone increased IL-10 and IL-6, likely due to the previously reported miR-155-5p-related effect on TLR4-promoted NF-κB signaling (Figure 8A) (68). In contrast, miR-221-3p mimics alone significantly repressed IL-10 secretion, but also promoted the secretion of IL-6. Strikingly, miR-221-3p also induced the M1-specific cytokine IL-12, markedly more than miR-155-5p mimics. The combined transfection of both miRs revealed that the enhanced IL-10 secretion caused by miR-155-5p mimics was significantly suppressed, indicating a more dominant regulatory input of miR-221-3p compared to miR-155-5p on IL-10 production in M2-macrophages. By contrast, combined miR-221-3p/miR-155-5p exposure significantly increased IL-12 secretion in M2-macrophages pointing at a synergistic effect of both miRs on promoting a pro-inflammatory response in these cells. As a consequence, the combination of miR-221-3p/miR-155-5p mimics, significantly decreased the anti-inflammatory activity in M2-macrophages compared to miR-155-5p alone as measured by IL-10/IL-12 or IL-10/IL-6 ratios (Figure 8B).

Since we have found that counteracting the downregulation of miR-221-3p following TLR4-stimulation increases a pro-inflammatory response and diminishes the anti-inflammatory activity of M2-macrophages, we asked if further downregulation of miR-221-3p would act in an opposite manner. We therefore performed loss-of-function studies by suppressing miR-221-3p and miR-155-5p with specific inhibitors in M2-macrophages (Figure S6). In contrast to the performed mimic experiments, inhibition of miR-221-3p in combination with LPS-treatment exhibited mild, but significant repressive effects on pro-inflammatory cytokines IL-6 and IL-12, while there was no effect on IL-10 secretion (Figure S6A). Furthermore, M2-macrophages transfected with miR-155-5p inhibitor exhibited a similar inflammatory response as with miR-221-3p inhibitor. As consequence, inhibition of miR-221-3p and miR-155-5p both slightly increased the anti-inflammatory activity level of M2-macrophages (Figure S6B).

Our data therefore suggest a decisive impact of miR-221-3p on the cytokine secretion profile of M2-macrophages thereby regulating the balance of pro- vs. anti-inflammatory functions.

## Discussion

Macrophages play a crucial role in the pathogenesis of RA by their secretion of pro-inflammatory cytokines and chemokines thereby contributing to the chronic synovitis characteristic for RA. The local microenvironment may have a decisive influence on the balance of pro-inflammatory vs. anti-inflammatory macrophages, as these cells exhibit a high functional plasticity in response to various stimuli (11, 69). Indeed, there seems to exist a link between inflammatory diseases such as RA and a dysbalanced M1/M2 ratio (2, 14). The identification of factors determining the functional differentiation of macrophages is essential as the underlying molecular processes may be important for the pathogenesis of RA and potentially other inflammatory diseases. We therefore aimed to analyze the role of specific miRs in the plasticity of M1- and M2-macrophages and evaluated miR-221-3p and miR-155-5p, as they were found to be dysregulated in RA. In our study, we measured significantly increased miR-221-3p levels in synovial tissue and also in the synovial fluid of RA patients compared to OA and OIA, suggesting a local concentration of dysregulated miR-221-3p at sites of inflammation. Interestingly, miR-221-3p and miR-155-5p displayed a similar expression profile in RA synovial fluid and tissue. MiR-155-5p is a well-documented contributor to inflammatory conditions (27, 31, 33, 70, 71). We show that dysregulated miR-221-3p expression alone or in combination with increased miR-155-5p levels is affecting the functional M1/M2-balance *in vitro*. We also found that M1-macrophages exhibit higher endogenous expression of miR-221-3p and miR-155-5p compared to M2-macrophages suggesting the former as possible origin of the aberrant miR-levels. However, additional cell populations such as synovial fibroblasts may also contribute to the dysregulated miR expression as increased miR-221-3p has been shown in cultured synovial fibroblasts from RA compared to OA patients (34).

In contrast to functional investigations of miR-221-3p in cancer (37), there is only limited knowledge about the function and targets of miR-221-3p in the context of RA. Studies using synovial fibroblasts demonstrated that downregulation of miR-221-3p decreased migration, invasion, and TNF-α production in these cells upon LPS treatment (35). Furthermore, TNF-α induced secretion of miR-221-3p in exosomes from synovial fibroblasts was shown to affect osteoblast differentiation thereby contributing to the bone loss in a RA mouse model (72). In our study, miR-221-3p was downregulated in human macrophages upon TLR4-stimulation suggesting a regulatory control of miR-221-3p by the TLR4-pathway under homeostatic conditions. Counteracting the TLR4-mediated suppression of miR-221-3p by transfecting M2-macrophages with specific mimics led to an increased release of pro-inflammatory mediators IL-6 and IL-8 and conversely, to reduced secretion of anti-inflammatory IL-10 and CXCL13. These effects were not seen when signaling through TLR2 or TLR3 as well as not in M1-macrophages. Applying specific miR-221-3p inhibitors to TLR-4 activated M2-macrophages instead reduced these pro-inflammatory features, thereby further substantiating the role of miR-221-3p in modulating the inflammatory response and functional plasticity of M2-macrophages.

TLR4-stimulation was shown to activate JAK3/STAT3 in M2-macrophages to sustain an autocrine IL-10 feedback and inhibit pro-inflammatory cytokine production (65). Furthermore, JAK3 inhibition led to reduced IL-10 and increased pro-inflammatory cytokine secretion in macrophages (73, 74). These results are in agreement with our findings in M2-macrophages. We could show that miR-221-3p mimics led to a prominent repression of JAK3 protein resulting in reduced Y705-STAT3 phosphorylation and reduced IL-10 secretion. RNA-seq data from M2-macrophages treated miR-221-3p mimics showed a prominent downregulation of JAK3 and the JAK/STAT-pathway was listed in the GSEA as a significantly suppressed pathway. Ultimately, a direct interaction of miR-221-3p and the 3′UTR of JAK3 was shown in luciferase reporter assays establishing JAK3 as new direct target of miR-221-3p.

Based on these results we suggest that under homeostatic conditions the downregulation of miR-221-3p in M2-macrophages is necessary to obtain an anti-inflammatory cytokine/chemokine profile. Hence, downregulation of miR-221-3p by TLR4-stimulation relieves the suppression of JAK3 protein allowing JAK3 activation via TLR4-induced IL-10, which in turn is resulting in STAT3 phosphorylation and suppression of pro-inflammatory features (66).

Consequently, aberrant high miR-221-3p expression levels combined with abundant TLR4-stimuli as found in the RA synovium are capable of interfering with the JAK3/STAT3 pathway and shift M2-macrophages toward a pro-inflammatory M1-function. The secretion of the prototypic M1-cytokine IL-12 by M2-macrophages in presence of high miR-221-3p levels as well as the decreased IL-10/IL-6 and IL-10/IL-12 ratios are clearly indicating a switch toward a M1-like inflammatory phenotype. Interestingly, in M2-macrophages miR-221-3p is exhibiting a more decisive regulatory impact on the measured cytokines than the inflammation-promoting miR-155-5p. Thus, combined transfection of miR-221-3p and miR-155-5p mimics increased IL-12 but suppressed IL-10, comparable to miR-221-3p mimics alone. In concerted action, miR-221-3p suppresses miR-155-5p-promoted IL-10 on one side, and on the other side further pushes IL-12 secretion. We therefore suggest that dysbalanced miR-221-3p and miR-155-5p levels in the RA synovium, either caused by increased expression in synovial macrophages or by other synovial cell populations, could disturb the anti-inflammatory activity of a M2-like macrophage population and support the generation of a pro-inflammatory synovial milieu.

## Data Availability Statement

The datasets generated and analyzed during the current study are available from the corresponding author on request. RNA-seq data will be stored at NCBI/GEO, Accession No. GSE133527.

## Ethics Statement

The studies involving human participants were reviewed and approved by Ethikkommission Nordwest- und Zentralschweiz (EKNZ 2014-51). The patients/participants provided their written informed consent to participate in this study.

## Author Contributions

LQ, AT, and DK were involved in study concept and design, drafting, and revising the manuscript. LQ, AT, and EH were involved in the acquisition of data. JR analyzed the RNA-seq data and drafted the RNA-seq part of the manuscript. AL and JH were conducting the experiments related to the miR-target luciferase reporter assays. LQ, AT, EH, and DK were involved in the analysis and interpretation of data. All authors read and approved the final manuscript.

### Conflict of Interest

The authors declare that the research was conducted in the absence of any commercial or financial relationships that could be construed as a potential conflict of interest.

## Abbreviations

ELISA: enzyme-linked immunosorbent assay
ERK: extracellular signal-regulated kinase
FCS: fetal calf serum
GM-CSF: granulocyte macrophage colony-stimulating factor
HD: Healthy donor
HEPES: 4-(2-hydroxyethyl)-1-piperazineethanesulfonic acid
IRF3: interferon regulatory factor 3
JAK3: tyrosine-protein kinase JAK3
LPS: lipopolysaccharide
MAPK: mitogen-activated protein kinase
M-CSF: macrophage colony-stimulating factor
miR: microRNA
NF-κB: nuclear factor kappa B
OA: osteoarthritis
OIA: other inflammatory diseases
Pam3: Pam3CysSerLys4
PBMC: peripheral blood mononuclear cells
PolyIC: polyinosinic:polycytidylic acid
PsA: psoriatic arthritis
PVDF: polyvinylidene difluoride
qRT-PCR: quantitative real-time polymerase chain reaction
RA: rheumatoid arthritis
SAPK/JNK: stress-activated protein kinase/c-Jun-N-terminal kinase
TLR: toll-like receptor.
