# Supplementary material for: miR-221-3p Drives the Shift of M2-Macrophages to a Pro-Inflammatory Function by Suppressing JAK3/STAT3 Activation
Source: Front Immunol. 2020 Jan 27;10:3087. doi: 10.3389/fimmu.2019.03087 (PMC6996464; doi:10.3389/fimmu.2019.03087)
Supplement: Supplementary file 1 [file Data_Sheet_1.pdf]

Supplementary File 1

RNA-seq Analysis Method

Reads were mapped to the human hg38 genome assembly with STAR (version 2.5.2a_modified) (Dobin et al., 2013) with default parameters, except filtering out multimapping reads with more than 10 alignment locations (outFilterMultimapNmax =10) and filtering reads without evidence in the spliced junction table (outFilterType="BySJout"). All subsequent RNA-seq data analysis was performed using the R software (version 3.5).

Gene expression was quantified by the qCount function of the R Bioconductor package QuasR (version 1.18) (Gaidatzis et al., 2015) as the number of reads that started within any annotated exon of a gene from the UCSC knownGenes annotation (downloaded on 2017-04-03). Read and alignment quality was evaluated using the qQCReport function of QuasR.

The R Bioconductor package edgeR (version 3.24.3) (Robinson et al., 2010) was used for differential gene expression analysis. Between samples normalization was done using the TMM method (Robinson and Oshlack, 2010). Because the expression levels distribution differed extensively between unstimulated and LPS-stimulated samples, we normalized them and analyzed them separately subsequently. Only genes with CPM (counts per million reads mapped) values more than 1 in at least 4 samples were retained. A principal component analysis (PCA) was performed on the normalized log-transformed CPM values. The principal components most associated to the biological variable of interest are illustrated in **Figure S1** (PC1 associated to LPS treatment, PC2 to donor, and PC7 and 12 isolating best the miR-221-3p overexpression effect). Because the expression levels distribution differed extensively between unstimulated and LPS-stimulated samples, we renormalized them and analyzed them separately subsequently for differential expression of genes. An additive generalized linear model controlling for donor-specific differences was fitted to the raw counts (function glmFit), and the effects of miR-221-3p transfection was tested using likelihood ratio tests (function glmLRT). P-values were adjusted by controlling the false-discovery rate (Benjamini-Hochberg method) and genes with a FDR lower than 10% were considered differentially expressed.

For the heatmap in Figure 3A, the logCPM values were corrected for donor-specific effects for visualization purposes, using the function removeBatchEffect from the Bioconductor limma package (version 3.38.3).

The gene set enrichment analysis was performed with the function camera (Wu and Smyth, 2012) from the edgeR package (using the default parameter value of 0.01 for the correlations of genes within gene sets) using gene sets derived from the KEGG pathway database (Subramanian et al., 2005) a subset of the curated gene sets (c2 collection) of the Molecular Signature Database (MSigDB v6.0) (Liberzon et al., 2015). We considered only sets containing more than 10 genes and gene sets with a false discovery rate lower than 10% were considered significant.

References

Dobin, A., Davis, C.A., Schlesinger, F., Drenkow, J., Zaleski, C., Jha, S., et al. (2013). STAR: ultrafast universal RNA-seq aligner. *Bioinformatics* 29(1), 15-21. doi: 10.1093/bioinformatics/bts635.

Gaidatzis, D., Lerch, A., Hahne, F., and Stadler, M.B. (2015). QuasR: quantification and annotation of short reads in R. *Bioinformatics* 31(7), 1130-1132. doi: 10.1093/bioinformatics/btu781.

Liberzon, A., Birger, C., Thorvaldsdottir, H., Ghandi, M., Mesirov, J.P., and Tamayo, P. (2015). The Molecular Signatures Database (MSigDB) hallmark gene set collection. *Cell Syst* 1(6), 417-425. doi: 10.1016/j.cels.2015.12.004.

Robinson, M.D., McCarthy, D.J., and Smyth, G.K. (2010). edgeR: a Bioconductor package for differential expression analysis of digital gene expression data. *Bioinformatics* 26(1), 139-140. doi: 10.1093/bioinformatics/btp616.

Robinson, M.D., and Oshlack, A. (2010). A scaling normalization method for differential expression analysis of RNA-seq data. *Genome Biol* 11(3), R25. doi: 10.1186/gb-2010-11-3-r25.

Subramanian, A., Tamayo, P., Mootha, V.K., Mukherjee, S., Ebert, B.L., Gillette, M.A., et al. (2005). Gene set enrichment analysis: A knowledge-based approach for interpreting genome-wide expression profiles. *Proc Natl Acad Sci U S A* 102(43), 15545-15550. doi: 10.1073/pnas.0506580102.

Wu, D., and Smyth, G.K. (2012). Camera: a competitive gene set test accounting for inter-gene correlation. *Nucleic Acids Res* 40(17), e133. doi: 10.1093/nar/gks461.

Supplementary Figures

A

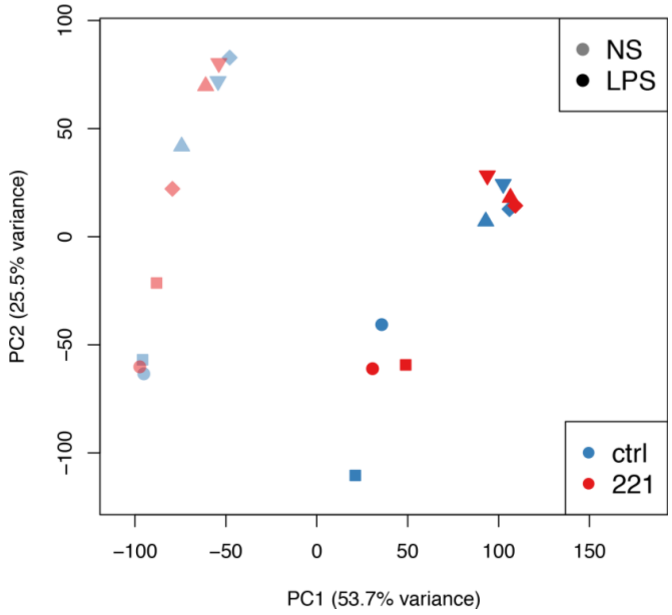

B

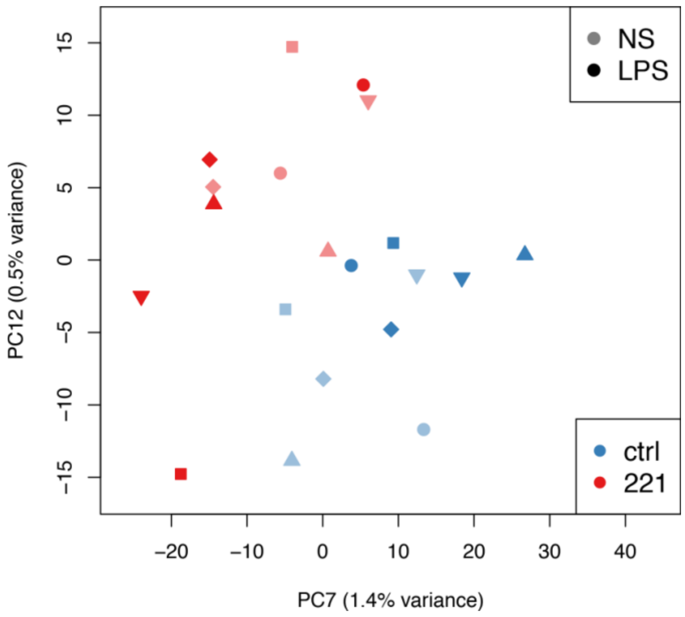

Figure S1. (A) Projection of RNA-seq samples on the first and second components of a principal component analysis. The dot color represents transfected cells samples (red) vs. controls cells samples (blue). The dot transparency represents stimulated vs. unstimulated samples. The dot shape represents the different donors. **(B)** Projection of RNA-seq samples on the seventh and twelfth components, most associated to the transfection effect.

LPS stimulation has an overall large impact on the transcriptome of M2-macrophages, unstimulated samples separated from stimulated sample on the first principal component of a principal component analysis, explaining more than half of the variance in the data. The PCA indicated that the transfection of miR-221-3p mimic instead had minor effect as it was only associated to deeper components (PC7 and 12 explaining respectively 1.4% and 0.5% of the variance). This was confirmed by the low number of differentially expressed genes between transfected and control cells in unstimulated conditions (8 genes showed significantly higher expression in miR-221-3p transfected cells, and 23 genes were downregulated) and after LPS stimulation (255 were upregulated and 132 genes were downregulated),

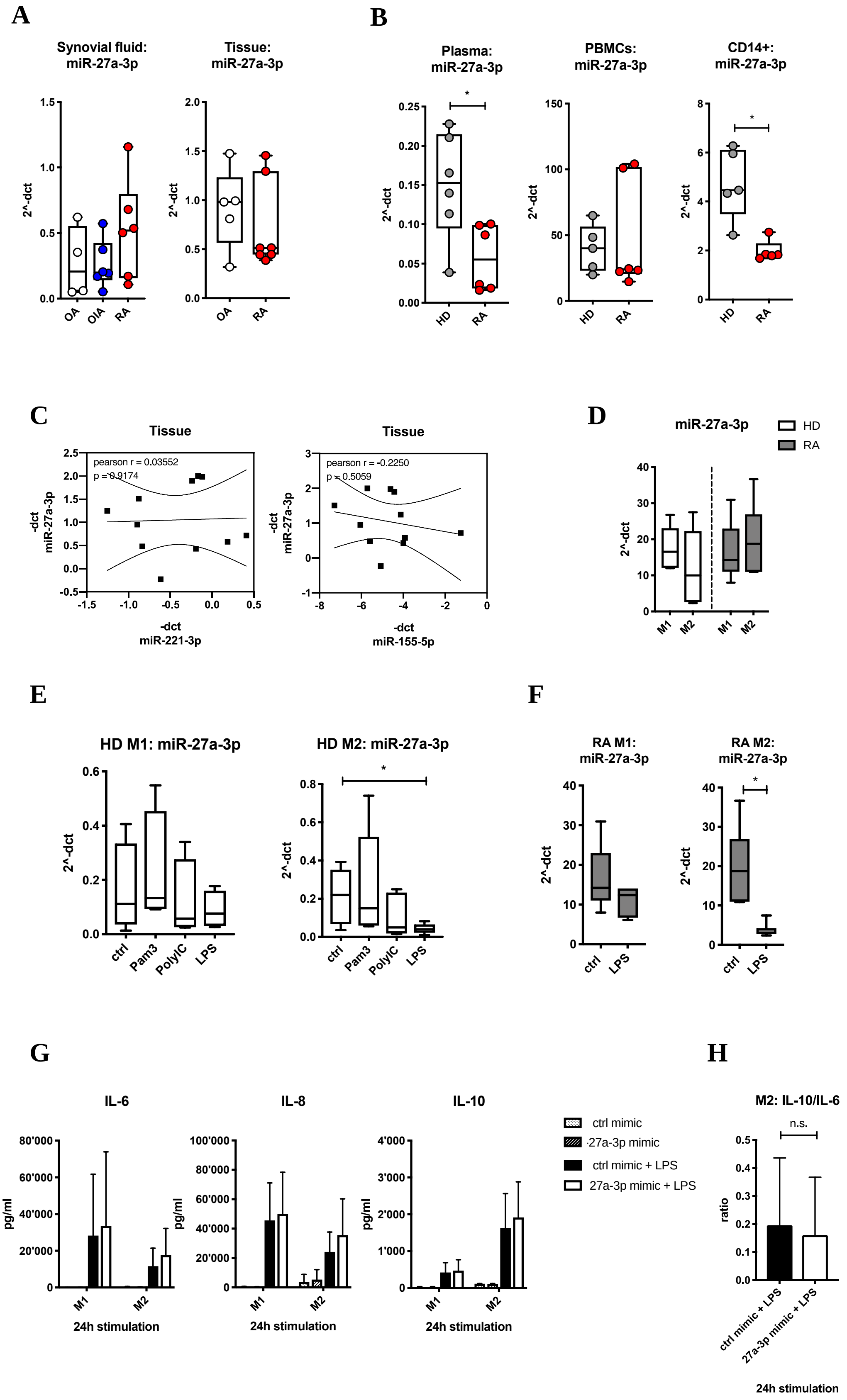
A
Synovial fluid: miR-27a-3p
Tissue: miR-27a-3p
2^-dct
OA
OIA
RA
B
Plasma: miR-27a-3p
PBMCs: miR-27a-3p
CD14+: miR-27a-3p
HD
RA
C
Tissue
pearson r = 0.03552
p = 0.9174
-dct miR-27a-3p
-dct miR-221-3p
pearson r = -0.2250
p = 0.5059
-dct miR-155-5p
D
miR-27a-3p
HD
RA
M1
M2
E
HD M1: miR-27a-3p
HD M2: miR-27a-3p
ctrl
Pam3
PolyIC
LPS
F
RA M1: miR-27a-3p
RA M2: miR-27a-3p
G
IL-6
IL-8
IL-10
pg/ml
24h stimulation
ctrl mimic
27a-3p mimic
ctrl mimic + LPS
27a-3p mimic + LPS
H
M2: IL-10/IL-6
n.s.
ratio
ctrl mimic + LPS
27a-3p mimic + LPS
24h stimulation

Figure S2. Results of miR-27a-3p. (A) miR-27a-3p endogenous expression level in the synovial fluid of RA patients was compared to OIA (psoriatic- and spondyloarthritis) and OA patients, and miR-27a-3p expression in synovial tissue was compared between biopsy samples from RA and OA patients. **(B)** miR-27a-3p endogenous expression level was measured in plasma, PBMCs and isolated $CD14^{+}$ cells from the peripheral blood of RA patients and HD. miR levels were measured by qRT-PCR and normalized to control miRs (miR-16-5p, miR-103-5p, miR-15b-5p or spike-in cel-miR-39) and expressed as $2^{-\Delta CT}$ by boxplot with min/max whiskers. N=4-7, * $p<0.05$. **(C)** -ΔCT level of miR-27a-3p was plotted against miR-221-3p and miR-155-5p level from synovial tissue and the Pearson correlation and p-values were computed. Data are plotted with regression coefficients (solid line) and 95% confidence intervals (broken lines). **(D)** Endogenous expression level of miR-27a-3p was compared in M1- and M2-macrophages generated from CD14+ cells of PBMCs from HD or RA patients and presented as $2^{-\Delta CT}$ by boxplot with min/max whiskers. N=3-6, * $p<0.05$. **(E)** Macrophages differentiated from CD14+ cells from HD or **(F)** RA blood were stimulated with either 300 ng/ml Pam3 (TLR2), 10 µg/ml PolyIC (TLR3) or 100 ng/ml LPS (TLR4) for 24 hours. Changes in miR-27a-3p expression level were measured by qRT-PCR. Values were normalized to RNU48 or miR-103a-3p and presented as $2^{-\Delta CT}$ by boxplot with min/max whiskers. N=3-6, * $p<0.05$ compared to untreated cells (ctrl). **(G)** Effects on cytokines and chemokines by miR-27a-3p in M1- and M2-macrophages. M1- and M2-macrophages were transfected with mimic of miR-27a-3p or a respective control miR and stimulated with 100 ng/ml LPS or left untreated. Secreted cytokines were measured after 24 hours by ELISA and values are expressed as mean ± S.D. N=4-7, * $p<0.05$. **(H)** Anti-inflammatory activity of generated M2-macrophages was calculated after 24 hours stimulation with LPS by the ratio of secreted IL-10 to IL-6.

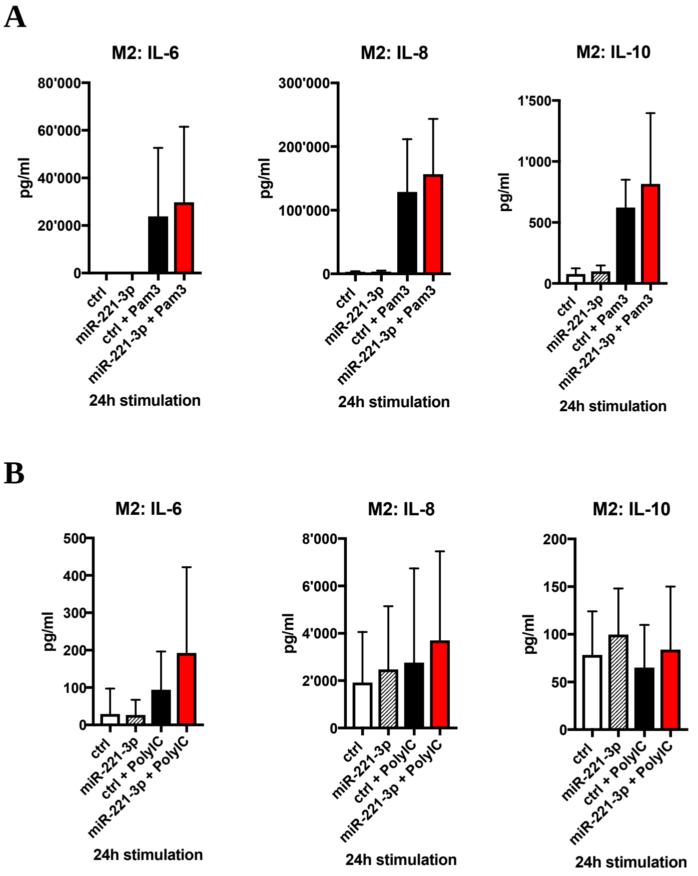

Figure S3. **Effects on cytokines and chemokines by miR-221-3p in TLR2- and TLR3-stimulated M2-macrophages.** M2-macrophages differentiated from CD14+ cells of HD blood were transfected with mimic of miR-221-3p or a respective control miR and stimulated with **(A)** 300 ng/ml Pam3 (TLR2 ligand) and **(B)** 10 µg/ml PolyIC (TLR3 ligand) or left untreated. Secreted cytokines were measured after 24 hours by ELISA and values are expressed as mean ± S.D. N=6-8, * $p<0.05$.

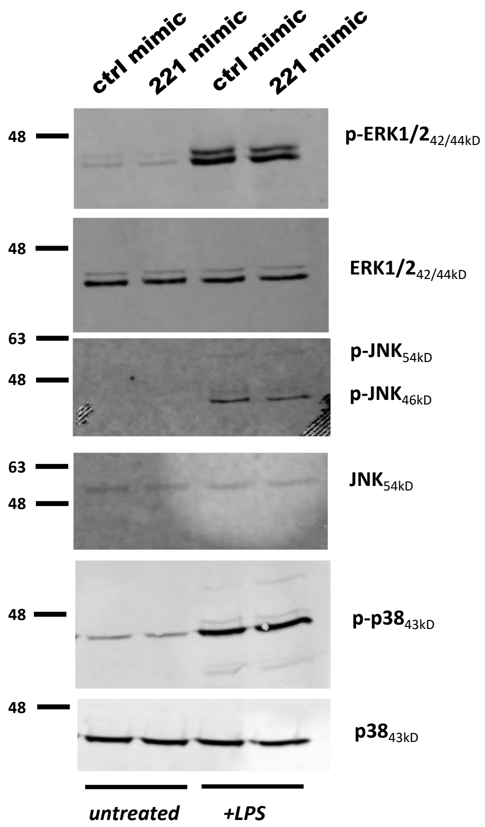

Figure S4. Effects of miR-221-3p mimics on MAPKs signaling in M2-macrophages. M2-macrophages differentiated from CD14+ cells of HD blood were transfected with miR-221-3p mimic or a respective control miR and stimulated with 100 ng/ml LPS for 30 minutes or left untreated. Proteins of whole cell extract were isolated and phosphorylation of MAPKs p38, ERK1/2 and c-Jun N-terminal kinase (JNK) was detected by Western Blot. N=2.

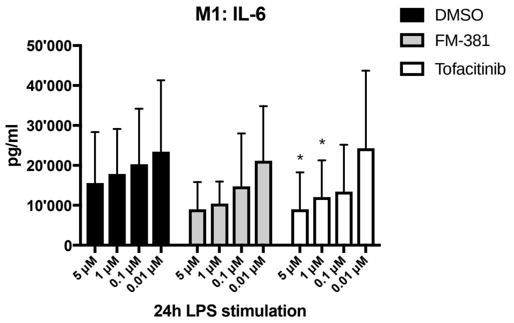
M1: IL-6
DMSO
FM-381
Tofacitinib
pg/ml
50'000
40'000
30'000
20'000
10'000
0
5 µM
1 µM
0.1 µM
0.01 µM
24h LPS stimulation

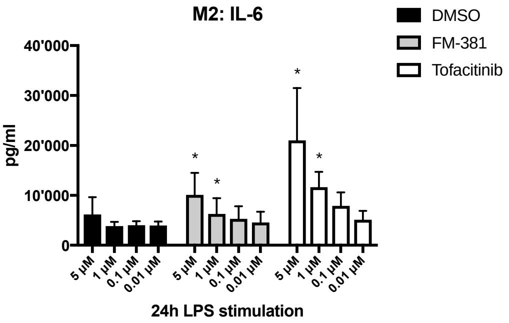
M2: IL-6
DMSO
FM-381
Tofacitinib
pg/ml
40'000
30'000
20'000
10'000
0
5 µM
1 µM
0.1 µM
0.01 µM
24h LPS stimulation

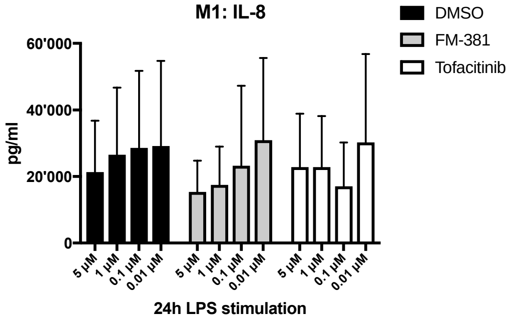
M1: IL-8
DMSO
FM-381
Tofacitinib
pg/ml
60'000
40'000
20'000
0
5 µM
1 µM
0.1 µM
0.01 µM
24h LPS stimulation

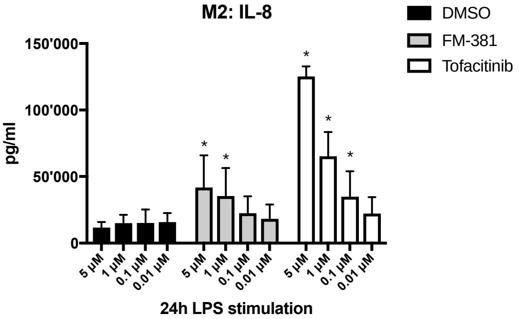
M2: IL-8
DMSO
FM-381
Tofacitinib
pg/ml
150'000
100'000
50'000
0
5 µM
1 µM
0.1 µM
0.01 µM
24h LPS stimulation

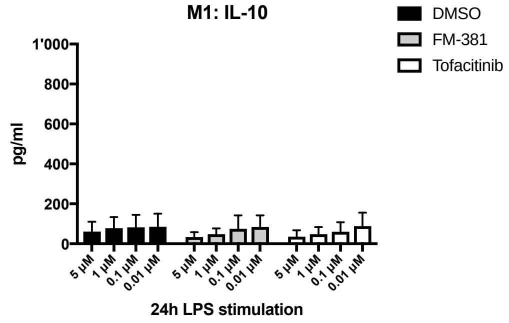
M1: IL-10
DMSO
FM-381
Tofacitinib
pg/ml
1'000
800
600
400
200
0
5 µM
1 µM
0.1 µM
0.01 µM
24h LPS stimulation

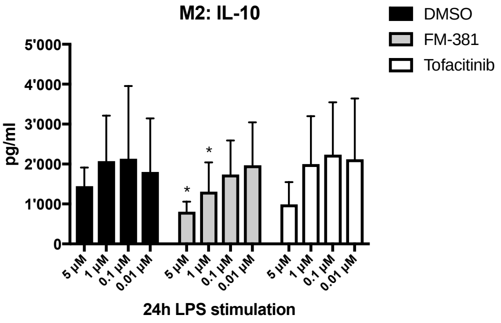
M2: IL-10
DMSO
FM-381
Tofacitinib
pg/ml
5'000
4'000
3'000
2'000
1'000
0
5 µM
1 µM
0.1 µM
0.01 µM
24h LPS stimulation

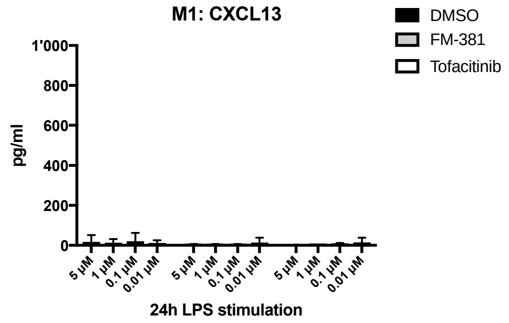
M1: CXCL13
DMSO
FM-381
Tofacitinib
pg/ml
1'000
800
600
400
200
0
5 µM
1 µM
0.1 µM
0.01 µM
24h LPS stimulation

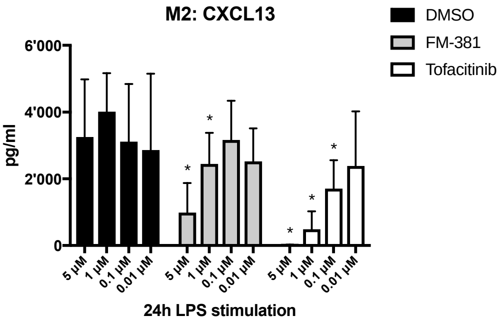
M2: CXCL13
DMSO
FM-381
Tofacitinib
pg/ml
6'000
4'000
2'000
0
5 µM
1 µM
0.1 µM
0.01 µM
24h LPS stimulation

Figure S5. Dose-dependent effect of JAK inhibition on macrophage subtypes. JAK3 function was inhibited in M1- and M2-macrophages generated from CD14+ cells from HD blood. Cells were treated with 5 µM, 1 µM, 0.1 µM or 0.01 µM JAK inhibitors FM-381 and Tofacitinib or DMSO control 1 hour prior to stimulation with 100 ng/ml LPS. Secreted cytokines were measured after 24 hours by ELISA and values are expressed as mean ± S.D. N=3-8, * $p<0.05$.

A

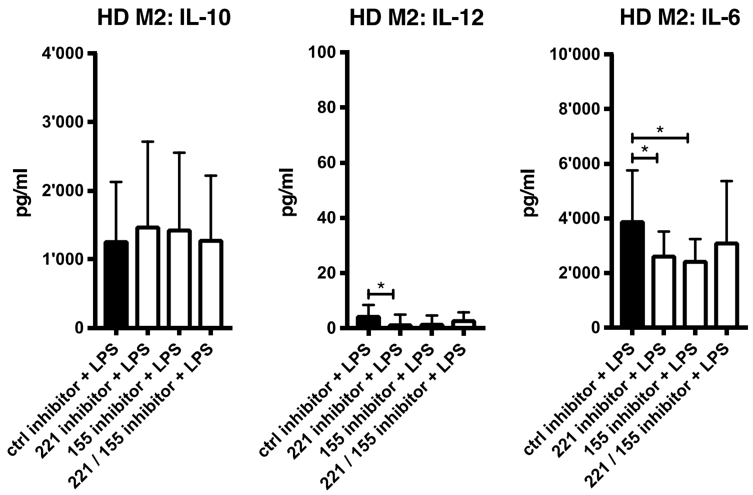

B

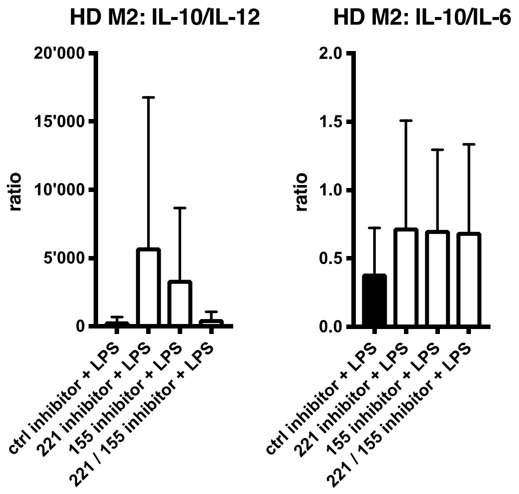

Figure S6. Loss-of function studies by transfection of miR-221-3p and miR-155-5p inhibitors in M2-macrophages. M2-macrophages differentiated from CD14+ cells of HD blood were transfected with inhibitors of miR-155-5p and miR-221-3p, either alone (155 inhibitor or 221 inhibitor) or combined (155 / 221 inhibitor) and a control miR (ctrl inhibitor). **(A)** M2-macrophages were stimulated with 100 ng/ml LPS or left untreated. Secreted cytokines were measured after 24 hours by ELISA and values are expressed as mean ± S.D. N=5, * $p<0.05$. **(B)** Anti-inflammatory activity of generated M2-macrophages transfected with the mentioned inhibitor combination setup was calculated after 24 hours LPS-stimulation by the ratio of secreted cytokines IL-10 to IL-12 or IL-10 to IL-6. Values are expressed as mean ± S.D. N=5, * $p<0.05$.
